# Retraction Note: Effects of waste glass and waste marble on mechanical and durability performance of concrete

**DOI:** 10.1038/s41598-023-39449-z

**Published:** 2023-08-01

**Authors:** Jawad Ahmad, Fahid Aslam, Rebeca Martinez‑Garcia, Jesús de‑Prado‑Gil, Shaker M. A. Qaidi, Ameni Brahmia

**Affiliations:** 1 Department of Civil Engineering, Military College of Engineering (NUST), Risalpur, 47040 Pakistan; 2 Department of Civil Engineering, College of Engineering in Al‑Kharj, Prince Sattam Bin Abdulaziz University, 11942 Al Kharj, Saudi Arabia; 3 Department of Mining Technology, Topography, and Structures, University of León, Campus de Vegazana S/N, 24071 León, Spain; 4 Department of Civil Engineering, College of Engineering, University of Duhok, Duhok, Iraq; 5 Department of Chemistry, College of Science, King Khalid University, 61413 Abha, Saudi Arabia

Retraction of: *Scientific Reports* https://doi.org/10.1038/s41598-021-00994-0, published online 02 November 2021

The Editors have retracted this article.

After publication concerns were raised that the XRD spectra in Figure 8 are identical and that the data points in Figure 16 and Figure 18 are the same although they report the results of different experiments. The authors are unable to provide the original data for examination. In addition, an investigation by the Editors has shown inappropriate changes in authorship during the review process. The Editors no longer have confidence in the results and conclusions presented.

Jawad Ahmad disagrees with this retraction. Fahid Aslam, Jesús de-Prado-Gil and Shaker M.A.Qaidi did not respond to the correspondence from the Editors about this retraction. The Editors were not able to obtain current contact details for Rebeca Martinez-Garcia and Ameni Brahmia.

